# Mortality risk in relation to diet quality assessed by the 2023 nutri-score nutrient profiling model: a prospective analysis

**DOI:** 10.1007/s00394-026-03946-4

**Published:** 2026-03-24

**Authors:** Nadine Khoury, Jose Cándido Fernández-Cao, Noushin Mohammadifard, Miguel Ángel Martinez-González, Dolores Corella, Montserrat Fitó, Ramón Estruch, Lucas Tojal-Sierra, Enrique Gómez Gracias, Miquel Fiol, José Lapetra, Lluís Serra-Majem, Xavier Pintó, Zenaida Vázquez-Ruiz, Jose V. Sorli, Helmut Schröder, Jordi Salas-Salvadó, Nancy Babio

**Affiliations:** 1 Institut de Recerca Biomèdica Catalunya Sud (IRBCatSud), Reus, 43204 Spain; 2 Departament de Bioquímica I Biotecnologia, Alimentació, Nutrició, Desenvolupament i Salut Mental ANUT-DSM, Universitat Rovira i Virgili, Reus, 43204 Spain; 3 Centro de Investigación Biomédica en Red Fisiopatología de La Obesidad y Nutrición (CIBEROBN), Instituto de Salud Carlos III, Madrid, 28029 Spain; 4 Department of Nutrition and Dietetics, Faculty of Health Sciences, University of Atacama, Copiapó, Chile; 5 Isfahan Cardiovascular Research Center, Cardiovascular Research Institute, Isfahan University of Medical Sciences, Isfahan, Iran; 6 Department of Preventive Medicine and Public Health, School of Medicine, University of Navarra, Pamplona, Spain; 7 Department of Nutrition, Harvard T.H. Chan School of Public Health, Boston, MA USA; 8 Department of Preventive Medicine and Public Health, School of Medicine, University of Valencia, Valencia, Spain; 9 Unit of Cardiovascular Risk and Nutrition, Institut Hospital del Mar de Investigaciones Médicas Municipal d’Investigació Médica (IMIM), Barcelona, 08003 Spain; 10 Department of Internal Medicine, Institut d’Investigacions Biomèdiques August Pi Sunyer (IDIBAPS), Hospital Clinic, University of Barcelona, Barcelona, 08036 Spain; 11 Department of Cardiology, Hospital Universitario de Álava, Vitoria, Spain; 12 Preventive Medicine and Public Health, University of Malaga, Malaga, 29071 Spain; 13 Osakidetza Basque Health Service, Bioaraba Health Research Institute, Araba University Hospital, University of the Basque Country UPV/EHU, Vitoria-Gasteiz, 01006 Spain; 14 Department of Family Medicine, Research Unit, Distrito Sanitario Atención Primaria Sevilla, Sevilla, 41009 Spain; 15 Research Institute of Biomedical and Health Sciences (IUIBS), Canarian Health Service, University of Las Palmas de Gran Canaria & Centro Hospitalario Universitario Insular Materno Infantil (CHUIMI), Las Palmas de Gran Canaria, 35016 Spain; 16 Lipid and Vascular Risk Unit, Internal Medicine Service, Hospital Universitario de Bellvitge, L’Hospitalet de Llobregat, Spain; 17 Centro de Investigación Biomédica en Red Epidemiología y Salud Pública (CIBEResp), Instituto de Salud Carlos III, Madrid, 28029 Spain

**Keywords:** Food labelling, Nutrient profile, Mortality risk, Nutri-Score

## Abstract

**Background:**

The updated Nutri-Score nutrient profiling model (*u*NS-NPM), revised in 2023, aims to better align with dietary guidelines and improve health outcomes prediction. However, evidence assessing its validity and applicability remains limited, particularly in Spanish populations.

**Objective:**

To investigate the prospective association between diet quality, assessed using the *u*NS-NPM dietary index (DI), and the risk of all-cause and cause-specific mortality in older adults at high cardiovascular risk.

**Methods:**

A prospective analysis within the PREDIMED cohort, with 7,212 participants aged 55–80 years at high cardiovascular risk was conducted. Diet was assessed by validated food frequency questionnaires, and the *u*NS-NPM DI was computed to quantify overall dietary quality. Time-dependent Cox regression models were used to estimate hazard ratios (HRs) and 95% confidence intervals (CIs) for all-cause, cardiovascular, cancer, and other-cause mortality across quintiles of the average cumulative *u*NS-NPM DI, adjusting for relevant confounders.

**Results:**

Over a median follow-up of 6 years, 425 deaths occurred (103 cardiovascular, 169 cancers, 153 other causes). Participants in the highest quintile of the uNS-NPM DI (reflecting poorer diet quality) had a higher risk of all-cause mortality (HR: 1.64; 95% CI: 1.19–2.28; p-trend = 0.007) and a higher risk of cardiovascular mortality (HR: 3.21; 95% CI: 1.29–7.95; p-trend = 0.002) compared to those participants in the lowest quintile. Participants in the highest quintile of uNS-NPM DI had also an increased risk of death from other causes (HR: 1.84; 95% CI: 1.11–3.07), although the trend was not statistically significant p-trend = 169). For cancer mortality, no significant association was observed (HR for highest vs. lowest quintile: 1.16; 95% CI: 0.69–1.92 p-trend = 0.695).

**Conclusions:**

In this Mediterranean cohort of older adults at high cardiovascular risk, lower dietary quality, assessed with the *u*NS-NPM DI, was prospectively associated with higher risks of all-cause, cardiovascular, and other-cause mortality. These findings support the *u*NS-NPM DI as a valuable tool for diet quality assessment.

**Supplementary Information:**

The online version contains supplementary material available at 10.1007/s00394-026-03946-4.

## Introduction

Chronic diseases are the leading cause of mortality and morbidity worldwide, accounting for approximately 74% of all deaths worldwide, posing a significant and growing challenge to global health [1]. Dietary factors play a crucial role in the development and progression of non-communicable diseases [2]. Fortunately, unhealthy dietary patterns are a major modifiable risk determinant that can be addressed through public health interventions. In response to this need, many European countries have developed official dietary guidelines aimed at preventing diet-related chronic diseases. However, additional intervention at the point of purchase is necessary to support healthier food choices. Front-of-pack (FOP) labeling systems have been proposed as part of a broader strategy to promote healthier eating habits [3]. Emerging evidence suggests that FOP labeling can play a significant role in improving consumer understanding of nutritional quality. It also has the potential to encourage the selection and purchase of healthier foods, while urging food industries to reformulate their products [4]. This dual impact on both consumer behavior and industry practices has the potential to significantly reduce the prevalence of chronic diseases [4].

One of the most prominent FOP labels is the Nutri-Score label, which ranks the overall nutritional quality of food using five color-coded letters (from A, dark green, to E, dark orange) [5]. The Nutri-Score is based on a nutrient profiling system (NPS) initially developed by the UK Food Standards Agency (FSA-NPS) and later modified for applicability purposes in different countries (FSAm-NPS) [6]. In 2023, the Nutri-Score algorithm was revised, following recommendations from the Nutri-Score international scientific committee, to better align with current dietary guidelines and an updated Nutri-Score Nutrient Profiling Model Dietary Index (*u*NS-NPM DI) was created [7].

Nutri-Score is one of the most extensively studied FOP labels in Europe. However, current EU regulations on consumer information make the use of such labels, voluntary with implementation left to the discretion of food manufacturers (WHO, 2019). The EU’s Farm-to-Fork strategy, however, outlines a plan to revise this regulation, aiming to establish a standardized and mandatory FOP label across different countries.

The World Health Organization emphasizes that a crucial step in validating a FOP label is to confirm the effectiveness of its nutrient profiling system by associating diet quality to health outcomes [3]. Previous studies have shown that diets with a higher FSAm-NPS dietary index (indicative of lower nutritional quality) are associated with higher risk of unhealthy outcomes such as metabolic syndrome, weight gain and increased cardiovascular diseases (CVD) and mortality [8–11]. In addition, studies have shown associations between lower FSAm-NPS scores and reduced mortality risk [11–14]. However, studies were mostly limited to French populations, using the old version of Nutri-Score and with relatively few events. These limitations highlight the need for further studies across diverse populations.

A recent large prospective study among 345,533 participants from the European Prospective Investigation into Cancer and Nutrition study (EPIC, 1992–2010, 7 European countries), individuals who consumed diets with lower nutritional quality, as indicated by the *u*NS-NPM score, were found to have a higher risk of CVD overall and across several subtypes [15], however the association between the *u*NS-NPS and mortality remains unexplored.

Given the recent adoption of the *u*NS-NPM in 2023, this study aims to examine the association between diet quality, assessed with the updated 2023 NS-NPM DI, and all-cause mortality and cause-specific mortality including CVD mortality, cancer mortality and death from other causes in an elderly population within the PREDIMED study cohort.

## Methodology

### Participants and design

For the present analysis, we used an observational approach in the PREDIMED trial (ISRCTN35739639), treating participants as a cohort, to assess associations between dietary nutritional quality and mortality. The trial’s methodology has been previously detailed [16, 17]. Briefly, between 2003 and 2009, a total of 7,447 participants men aged 55–80 and women aged 60–80 were enrolled. Although they had no CVD at baseline, all participants were considered at high risk. Eligibility required either a diagnosis of type 2 diabetes or the presence of at least three CVD risk factors, such as high cholesterol, low HDL cholesterol, overweight or obesity, hypertension, smoking, or a family history of early-onset coronary heart disease. Individuals were excluded if they had alcohol or drug dependency, a serious chronic illness, a body mass index (BMI) ≥ 40 kg/m², or allergies/intolerances to olive oil or nuts. Participants were randomly assigned to one of three groups: a Mediterranean diet (MedDiet) supplemented with extra-virgin olive oil, a MedDiet supplemented with mixed nuts, or a control group that received guidance to reduce animal and vegetable fat intake [17]. This analysis includes an extended observational follow-up of mortality through June 30, 2012. The PREDIMED study received approval from the institutional review boards of participating centers, and all participants gave written informed consent.

### Dietary intake assessement and uNS-NPM DI computation

Dietary intake was evaluated at baseline and yearly using a validated food frequency questionnaire (FFQ) [18], conducted by trained dietitians. Participants reported their habitual intake over the preceding year, specifying the frequency and portion size of 137 food and beverage items. Consumption frequency was recorded using a nine-level scale, ranging from “never or almost never” to “more than six times per day,” with standardized portion sizes (e.g., slices, glasses, teaspoons) provided to ensure consistency in reporting. Nutrient intake was estimated by multiplying the reported daily frequency of consumption for each item by its corresponding nutrient content, as defined in the Spanish food composition database [19]. Total energy intake was also estimated from the quantity and frequency of food and beverage consumption.

Each food and beverage item in the PREDIMED food composition database [19] based on generic food categories rather than branded products, was assigned a uNS-NPM score. The database does not include commercial branded items; therefore, nutrient profiling was performed using generic nutrient compositions. The method used to calculate this score is described in the Supplementary Material and detailed in previous publications [7]. In summary, the score is based on the nutritional content per 100 g of food or per 100 milliliters of beverages. Points are given according to components that should be limited: sugars, saturated fats, salt, and energy and according to beneficial components: dietary fiber, protein, and percentage of fruits, vegetables, and pulses. The final *u*NS-NPM score is calculated by subtracting the total points that should be limited from the total beneficial points. This score ranges from − 17 to + 55, with higher scores indicating lower nutritional quality.

To evaluate an individual’s diet quality using these food-level scores, a dietary index (uNS-NPM DI) was calculated [7]. This individual-level score was calculated by summing the *u*NS-NPM scores of all consumed foods and drinks, weighted by their energy contribution (calculated using the energy content per 100 g and estimated daily intake), and then dividing by the person’s total daily energy intake following this equation:

1 $$\mathrm{D}\mathrm{i}\mathrm{e}\mathrm{t}\mathrm{a}\mathrm{r}\mathrm{y}\;\mathrm{I}\mathrm{n}\mathrm{d}\mathrm{e}\mathrm{x}=\frac{\sum_{i=1}^{n}NSiEi}{{\sum}_{i=1}^{n}Ei}$$

Where “i” signifies a food or beverage consumed by the participant, “NSi” thefood (or beverage) score, “Ei”, the mean daily energy intake from this food (orbeverage) and “n” the number of different foods.

Therefore, higher *u*NS-NPM DI scores reflects lower nutritional quality of the individual’s overall diet.

To minimize the random measurement error caused by within-person variation and to better represent the long-term nutritional quality, we used the cumulative average from baseline to the last FFQ before death using all available FFQ data until their participation in the PREDIMED trial ended. All nutrient and food intakes were adjusted for total energy intake using the residual method [20].

### Mortality and cause of death ascertainment

The endpoints assessed in this study included: (1) all-cause mortality, (2) CVD mortality, (3) cancer-related mortality, and (4) mortality due to other causes, as determined within the PREDIMED trial [17]. Mortality data were systematically updated on an annual basis by an endpoint adjudication committee that was blinded to participants’ dietary intake and intervention group allocation. Mortality ascertainment was based on multiple sources: annual participant questionnaires and clinical assessments, direct communication with primary care physicians, annual reviews of medical records, and data linkage with the Spanish National Death Index. For this analysis, only deaths occurring between June 25, 2003, and June 30, 2012, were considered.

### Covariate ascertainment

At the time of enrollment, information was collected on sociodemographic, lifestyle habits, body measurements, medical history, and medication use. Participants self-reported the family history of CVD or cancer, or if they had been previously diagnosed with type 2 diabetes, hypercholesterolemia, or hypertension. Medication use was confirmed to determine whether participants were receiving treatment for these conditions. Physical activity was assessed using a validated Spanish version of the Minnesota Leisure Time Physical Activity Questionnaire [21]. Adherence to the MedDiet was measured annually using a validated 14-item questionnaire [22]. Height and weight were measured by trained staff and the BMI was then calculated as weight divided by height squared (kg/m2).

### Statistical analysis

Descriptive statistics were used to summarize baseline characteristics of the study population overall and across quintiles of *u*NS-NPM DI. Continuous variables were expressed as means and SD and compared across quintiles using one-way ANOVA. Categorical variables were presented as frequencies and percentages, and differences across quintiles were assessed using the chi-square test.

Time-dependent Cox regression models were used to assess the associations between the *u*NS-NPM DI in quintile and in continuous with 1 standard deviation (SD) increment, and mortality outcomes including all-cause mortality, CVD mortality, cancer mortality, and mortality due to other causes. Time-to-event was calculated from baseline to the date of death or date of the last vital information obtained. Hazard ratios (HRs) and 95% confidence intervals (CIs) were estimated across quintiles of the *u*NS-NPM DI, using the lowest quintile (Q1) as the reference group. Two models were employed: Minimally-adjusted model: adjusted for age (continuous, in years) and sex (male/female) and fully-adjusted model: further adjusted for total energy intake (kcal/day, continuous), intervention group (olive oil, nuts, low-fat diet), education level (primary, secondary, university), smoking status (never, former, current), physical activity (MET-minutes/day, continuous), body mass index (underweight/normal weight or overweight/obesity), alcohol consumption (g/day, continuous), family history of cancer (yes/no), prevalence of diabetes (yes/no), hypertension (yes/no) and hypercholesterolemia (yes/no).

Linear trend tests across quintiles were conducted by modeling the median value of each quintile as a continuous variable.

Tests for interactions between the *u*NS-NPM DI and mortality outcomes were performed using the likelihood ratio tests and multivariable Cox proportional hazards regression models were then conducted to assess the stratification and subgroup analyses of the following variables: sex (male, female), education level (primary, secondary, university) BMI (</≥ 25 kg/m2), age (</≥67; median value), adherence to MedDiet (</≥7; median value) and ultra-processed food consumption (cumulative average, energy-adjusted in g/day; median value); results of the interaction tests and corresponding subgroup analyses are shown in Supplementary Material File S2. Robust variance estimators were used to account for intra-cluster correlations. Additionally, we performed a sensitivity analysis by excluding participants who experienced a mortality event within the first one or two-year of follow-up, consistent with previous studies [23]. This approach aimed to reduce the possibility of reverse causality bias, since individuals with a higher baseline risk of cardiovascular disease might have reported healthier dietary habits due to recent behavior changes triggered by a diagnosis before the baseline assessment (Supplementary Material File S3-4). To account for competing events, we conducted competing-risks analyses using Fine–Gray models, with results shown in the Supplementary Material File S5. Moreover, as a sensitivity analysis, we conducted additional models evaluating the association between uNS-NPM DI at baseline (with 1 SD increment) and cause-specific and all-cause mortality; results are shown in the Supplementary Material File S6.

All statistical tests were performed using Stata (version 18, StataCorp, USA) and 2-tailed P-value < 0.05 was considered statistically significant.

## Results

Baseline characteristics and dietary intake of the study population are shown in Table 1. The study cohort included 7,212 individuals with a mean age of 67.0 years (6.2), of whom 42.5% were male and 57.5% female. We excluded participants with missing dietary data (*n* = 78), who reported implausible energy intakes < 500 or > = 3500 kcal/d for women and < 800 or > = 4000 kcal/d for men [20]) at baseline (*n* = 153) or without any follow-up (*n* = 4). The mean *u*NS-NPM DI scores ranged from 1.4 in Q1 (healthiest diet) to 5.9 in Q5 (least healthy diet). Participants in the lowest quintile (Q1) had lower educational attainment, with 82.0% reporting primary-level education, compared to 74.1% in Q5. Compared to participants in the lowest quintile, those in the highest quintile presented higher prevalence of current smokers. In addition, participants with higher *u*NS-NPM DI (i.e., less favorable diets) were more likely to consume lower amounts of vegetables, fruits, legumes, nuts, fish, dairy products, and extra virgin olive oil. Conversely, participants with higher *u*NS-NPM DI scores, were more likely to consume ultra-processed foods, and red meat. Moreover, individuals in Q5 (meaning lower nutritional quality) were more likely to present higher total energy and carbohydrate intake, and a lower intake of protein and fiber.

**Table 1:** Baseline characteristic of the study population overall and across quintiles of *u*NS-NPM DI (*n* = 7,212)

		Quintiles of uNS-NPM DI				
	Total (*n* = 7,212)	1 (*n* = 1,443)	2 (*n* = 1,442)	3 (*n* = 1,443)	4 (*n* = 1,442)	5 (*n* = 1,442)
*u*NS-NPM DI (cumulative)	3.6 (1.6)	1.4 (0.7)	2.7 (0.2)	3.5 (0.2)	4.4 (0.3)	5.9 (0.9)
Sex						
Male	3,068 (42.5%)	482 (33.4%)	544 (37.7%)	601 (41.6%)	674 (46.7%)	767 (53.2%)
Female	4,144 (57.5%)	961 (66.6%)	898 (62.3%)	842 (58.4%)	768 (53.3%)	675 (46.8%)
Age (years)	67.0 (6.2)	67.3 (5.9)	67.5 (6.0)	67.2 (6.2)	66.8 (6.4)	66.3 (6.4)
Intervention group						
Olive oil	2,474 (34.3%)	495 (34.3%)	543 (37.7%)	503 (34.9%)	507 (35.2%)	426 (29.5%)
Nuts	2,359 (32.7%)	568 (39.4%)	489 (33.9%)	486 (33.7%)	425 (29.5%)	391 (27.1%)
Low fat diet	2,379 (33.0%)	380 (26.3%)	410 (28.4%)	454 (31.5%)	510 (35.4%)	625 (43.3%)
Education level						
Primary	5,603 (77.7%)	1,183 (82.0%)	1,153 (80.0%)	1,134 (78.6%)	1,064 (73.8%)	1,069 (74.1%)
Secondary	1,095 (15.2%)	179 (12.4%)	183 (12.7%)	207 (14.3%)	268 (18.6%)	258 (17.9%)
University	514 (7.1%)	81 (5.6%)	106 (7.4%)	102 (7.1%)	110 (7.6%)	115 (8.0%)
Smoking status						
Never	4,436 (61.5%)	1,013 (70.2%)	962 (66.7%)	905 (62.7%)	840 (58.3%)	716 (49.7%)
Former	1,004 (13.9%)	120 (8.3%)	152 (10.5%)	188 (13.0%)	225 (15.6%)	319 (22.1%)
Current	1,772 (24.6%)	310 (21.5%)	328 (22.7%)	350 (24.3%)	377 (26.1%)	407 (28.2%)
Physical activity METs min/day	234.0 (247.1)	253.1 (247.1)	234.5 (227.3)	234.4 (239.6)	227.8 (264.8)	219.9 (254.4)
BMI (kg/m^2^)	30.0 (3.9)	29.7 (3.9)	29.8 (3.8)	30.1 (3.8)	30.2 (3.9)	30.0 (3.9)
Family history of CVD						
No	5,433 (75.3%)	1,090 (75.5%)	1,082 (75.0%)	1,078 (74.7%)	1,094 (75.9%)	1,089 (75.5%)
Yes	1,779 (24.7%)	353 (24.5%)	360 (25.0%)	365 (25.3%)	348 (24.1%)	353 (24.5%)
Family history of cancer						
No	3,201 (44.4%)	626 (43.4%)	640 (44.4%)	641 (44.4%)	654 (45.4%)	640 (44.4%)
Yes	4,011 (55.6%)	817 (56.6%)	802 (55.6%)	802 (55.6%)	788 (54.6%)	802 (55.6%)
Diabetes prevalence						
No	3,686 (51.1%)	651 (45.1%)	675 (46.8%)	741 (51.4%)	777 (53.9%)	842 (58.4%)
Yes	3,526 (48.9%)	792 (54.9%)	767 (53.2%)	702 (48.6%)	665 (46.1%)	600 (41.6%)
Hypertension prevalence						
No	1,245 (17.3%)	272 (18.8%)	243 (16.9%)	226 (15.7%)	257 (17.8%)	247 (17.1%)
Yes	5,967 (82.7%)	1,171 (81.2%)	1,199 (83.1%)	1,217 (84.3%)	1,185 (82.2%)	1,195 (82.9%)
Hypercholesterolemia prevalence						
No	2,003 (27.8%)	363 (25.2%)	392 (27.2%)	401 (27.8%)	429 (29.8%)	418 (29.0%)
Yes	5,209 (72.2%)	1,080 (74.8%)	1,050 (72.8%)	1,042 (72.2%)	1,013 (70.2%)	1,024 (71.0%)
Adherence to Mediterranean diet (MEDAS score: 0–14 points)	5.8 (4.4)	5.9 (4.7)	6.1 (4.5)	5.7 (4.5)	5.9 (4.2)	5.2 (4.0)
Dietary intake						
Vegetables (g/day)	334.0 (144.5)	389.4 (163.7)	348.0 (142.5)	333.6 (133.5)	315.8 (132.2)	283.5 (126.0)
Fruits (g/day)	368.3 (195.7)	444.1 (211.6)	398.6 (190.8)	370.0 (191.4)	338.6 (177.2)	290.3 (169.5)
Cereals (g/day)	225.2 (82.6)	229.6 (73.4)	231.2 (77.8)	228.8 (81.3)	224.1 (86.2)	212.3 (91.7)
Legumes (g/day)	20.6 (13.3)	24.0 (16.5)	21.6 (13.6)	20.9 (13.4)	19.1 (10.7)	17.3 (10.2)
Extra virgin olive oil (g/day)	39.1 (16.7)	42.7 (15.6)	41.3 (16.7)	40.2 (16.6)	38.8 (16.6)	32.3 (16.2)
Nuts (g/day)	10.1 (13.1)	15.2 (16.2)	11.8 (12.7)	10.0 (11.7)	8.1 (11.8)	5.4 (10.4)
Dairy products (g/day)	380.3 (215.9)	414.4 (214.3)	403.2 (222.6)	380.8 (211.0)	358.9 (214.0)	344.1 (209.7)
Fish and seafood (g/day)	99.2 (48.9)	111.2 (47.1)	104.5 (56.8)	98.5 (42.8)	96.7 (46.0)	85.3 (46.8)
Red meat(g/day)	50.3 (35.2)	47.4 (31.3)	49.6 (32.3)	50.7 (34.2)	51.4 (34.8)	52.5 (42.3)
Processed meat (g/day)	26.0 (18.4)	21.8 (13.9)	24.4 (15.8)	25.5 (16.1)	27.0 (17.1)	31.5 (25.3)
White meat (g/day)	54.6 (32.0)	61.9 (32.0)	57.9 (31.6)	53.7 (31.8)	51.8 (31.1)	47.8 (31.9)
Ultra-processed food (g/day)	296.6 (156.2)	225.3 (107.4)	261.4 (118.7)	293.5 (136.9)	315.2 (155.5)	387.9 (196.3)
Total energy intake (kcal/day)	2236.0 (544.0)	1939.3 (444.5)	2116.4 (480.3)	2225.1 (494.7)	2362.9 (523.9)	2536.4 (569.3)
Total fat intake (g/day)	97.2 (17.1)	96.4 (16.1)	97.0 (16.3)	97.1 (16.8)	98.5 (17.2)	97.0 (18.7)
Total protein intake (g/day)	91.3 (14.0)	95.7 (13.0)	93.6 (13.9)	91.2 (13.3)	89.4 (13.6)	86.6 (14.2)
Total carbohydrate intake (g/day)	234.4 (40.8)	232.4 (37.0)	232.7 (37.6)	234.7 (40.2)	233.3 (42.0)	239.0 (46.3)
Fiber (g/day)	25.2 (7.5)	28.7 (7.4)	26.2 (7.2)	25.4 (7.2)	24.0 (7.2)	22.0 (6.9)
Alcohol (g/day)	8.3 (13.3)	8.0 (10.1)	8.3 (11.6)	8.3 (13.7)	8.4 (14.0)	8.6 (16.2)

uNS-NPM DI: updated Nutri-Score – Nutrient Profiling Model; BMI: body mass index

Data are expressed as Mean (SD) for continuous variable or frequency (%) for categoricalvariable using one-way ANOVA or chi-square respectively.

Associations between *u*NS-NPM DI across quintiles and cause-specific and all-cause mortality are shown in Table 2. During a median follow-up of 6 years (totaling 42,464 person-years), 425 deaths occurred, including 103 from CVD, 169 from cancer, and 153 from other causes.

**Table 2:** Association between NS-NPM DI and cause-specific and all-cause mortality; multivariable Cox proportional hazards regression models (*n* = 7,212)

	uNS-NPM DI in quintiles Hazard ratios (95% CI)					
	Q1 *n* = 1,**443**	Q2 *n* = 1,**442**	Q3 *n* = 1,**443**	Q4 *n* = 1,**442**	Q5 *n* = 1,**442**	*P*-trend
All-cause mortality *425 cases/42,464 py*	65 cases	77 cases	95 cases	84 cases	104 cases	
Mortality rate (per 1000)*	7.37	9.07	11.19	10.06	12.49	
Minimally-adjusted	1 (ref.)	1.17 (0.85 to 1.63)	**1.43 (1.04 to 1.96)**	1.20 (0.87 to 1.66)	**1.51 (1.11 to 2.05)**	**0.022**
Fully-adjusted	1 (ref.)	1.19 (0.86 to 1.66)	**1.51 (1.10 to 2.07)**	1.28 (0.91 to 1.79)	**1.64 (1.19 to 2.28)**	**0.007**
CVD mortality *103 cases/42,464 py*	7 cases	8 cases	30 cases	29 cases	29 cases	
Mortality rate (per 1000)*	0.79	0.94	3.53	3.47	3.48	
Minimally-adjusted	1 (ref.)	1.08 (0.39 to 2.98)	**3.93 (1.71 to 9.05)**	**3.56 (1.53 to 8.26)**	**3.69 (1.59 to 8.55)**	**0.002**
Fully-adjusted	1 (ref.)	1.00 (0.37 to 2.72)	**3.85 (1.67 to 8.88)**	**3.28 (1.38 to 7.80)**	**3.21 (1.29 to 7.95)**	**0.002**
Cancer *mortality* *169 cases/42,464 py*	32 cases	38 cases	33 cases	29 cases	37 cases	
Mortality rate (per 1000)*	3.63	4.48	3.88	3.47	4.44	
Minimally-adjusted	1 (ref.)	1.17 (0.73 to 1.86)	0.99 (0.61 to 1.61)	0.84 (0.51 to 1.39)	1.06 (0.66 to 1.71)	0.861
Fully-adjusted	1 (ref.)	1.19 (0.75 to 1.89)	1.03 (0.63 to 1.69)	0.91 (0.54 to 1.52)	1.16 (0.69 to 1.92)	0.695
Other causes of mortality *153 cases/42,464 py*	26 cases	31 cases	32 cases	26 cases	38 cases	
Mortality rate (per 1000)*	2.95	3.65	3.77	3.11	4.56	
Minimally-adjusted	1 (ref.)	1.23 (0.73 to 2.07)	1.24 (0.73 to 2.08)	0.97 (0.56 to 1.67)	1.42 (0.86 to 2.33)	0.418
Fully-adjusted	1 (ref.)	1.36 (0.79 to 2.30)	1.41 (0.84 to 2.38)	1.14 (0.64 to 2.05)	**1.84 (1.11 to 3.07)**	0.169

Multivariable Cox proportional hazards regression minimally adjusted model: adjusted for age (years, continuous) and sex (male, female). Fully-adjusted model: further adjusted for total energy intake (Kcal, continuous), intervention group (Olive oil, nuts, low fat diet), education level (primary, secondary, university), smoking status (never, former, current), physical activity (METS min/day, continuous), BMI (normal weight and overweight/obesity), alcohol consumption (g/day, continuous) family history of cancer (yes/no), diabetes (yes/no), hypertension (yes/no) and hypercholesterolemia (yes/no)

Abbreviations: *u*NS-NPM DI: updated Nutri-Score Nutrient Profiling Model Dietary Index; CVD: cardiovascular disease; HR: Hazard Ratios; CI: Confidence interval; SD: Standard Deviation

* failures/person-time (per 1000)

In fully-adjusted Cox regression models, compared to participants in the lowest quintile, those participants in the highest *u*NS-NPM DI quintiles had a higher risk of all-cause mortality (HR: 1.64; 95% CI: 1.19–2.28; p-trend = 0.007). Participants in the highest *u*NS-NPM DI quintile showed a higher risk of CVD mortality compared to participants in the lowest quintile (HR: 3.21; 95% CI: 1.29–7.95; p-trend = 0.002). In addition, individuals in the highest quintile of the *u*NS-NPM DI (Q5) had an increased risk of death from other causes compared to those in the lowest quintile (HR: 1.84; 95% CI: 1.11–3.07). No significant associations were observed for cancer mortality in the fully-adjusted model.

**Fig. 1:**
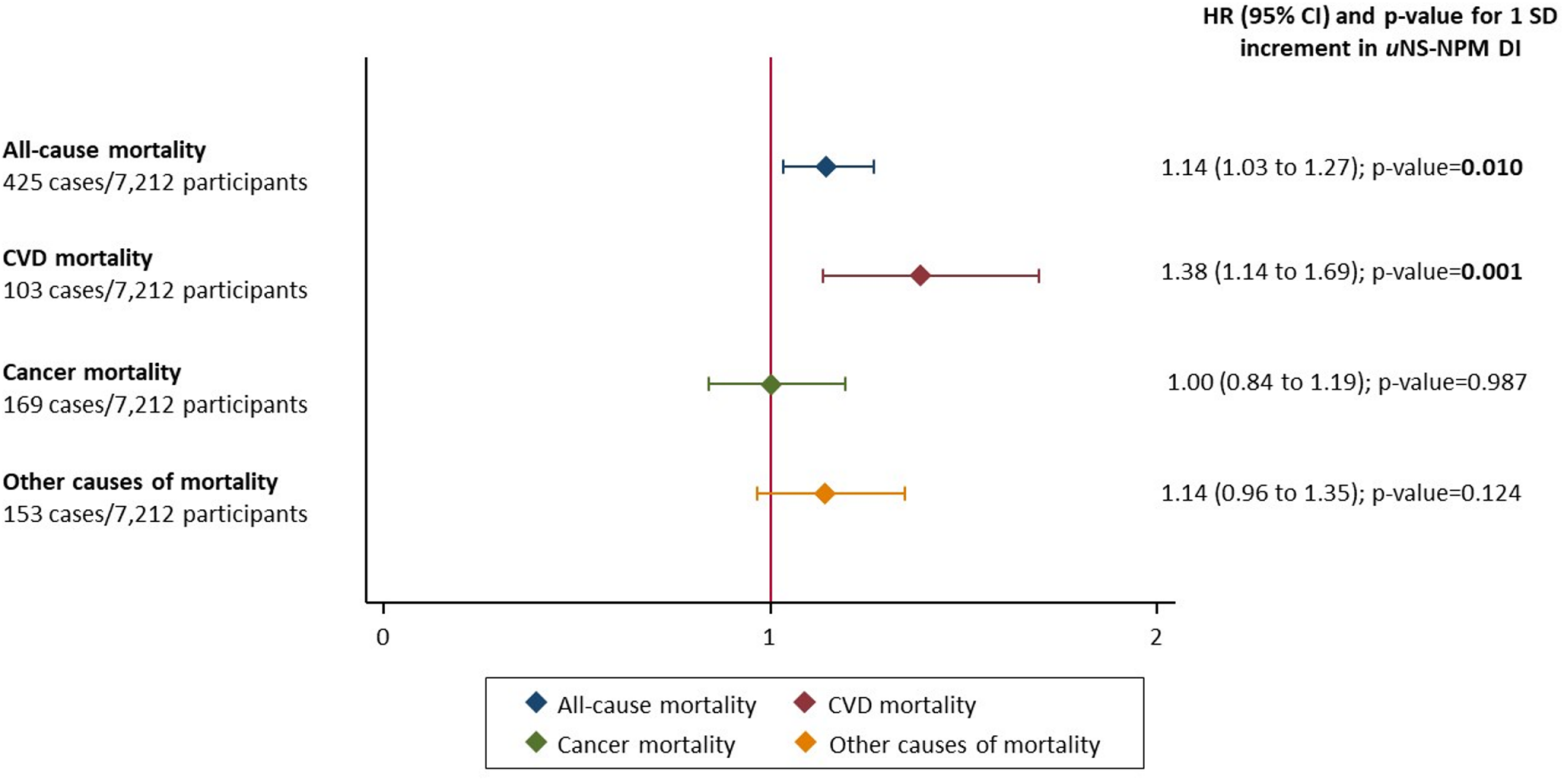
Association between *u*NS-NPM DI (for 1 SD increment) and cause-specific and all-cause mortality; multivariable Cox proportional hazards regression models (*n* = 7,212)

Associations between *u*NS-NPM DI (continuous, with 1 SD increment) and mortality risk are shown in Fig. 1. 1 SD increment in the *u*NS-NPM DI score was associated with a relatively higher rate of all-cause mortality (HR: 1.14; 95% CI: 1.03 to 1.27; p-value = 0.010) and relatively higher rate of CVD mortality (HR: 1.38; 95% CI: 1.14 to 1.69; p-value = 0.001). No significant associations were observed for cancer mortality nor for other causes of mortality. Interactions between the NS-NPS DI (1-SD increment) and other covariates in associations with mortality risk, and corresponding subgroup analyses are shown in **Supplementary Material File S2**. No interaction was observed with sex, education level, BMI and age, adherence to MedDiet and ultra-processed food consumption. When we excluded deaths during the first 1-year and, separately, during the first two years effect, estimates for all-cause and cardiovascular mortality remained consistent with the main analyses.

(**Supplementary Material File S2)**, the associations between higher uNS-NPM DI (reflecting lower nutritional quality) and increased all-cause and cardiovascular mortality risk remained robust. Similarly, no significant associations were observed for cancer mortality, while a positive association with mortality from other causes was shown after excluding early deaths within 2-year.

## Discussion

In this large prospective Mediterranean cohort, a higher *u*NS-NPM DI, indicating poorer diet quality, was associated with increased risks of all-cause, cardiovascular, and other-cause mortality. These associations remained statistically significant even after adjusting for a range of potential confounders.

Our results in relation to all-cause mortality align with previous studies linking nutrient profiling systems to all-cause mortality. For instance, in Spain, a higher baseline FSAm-NPS DI score was directly associated with all-cause mortality in the SUN study including 20,503 university graduates’ participants [12]. In the EPIC study including 501,594 adults with a median follow-up of 17.2 years, those participants with a higher FSAm-NPS DI score (highest versus lowest quintile) also showed an increased risk of all-cause mortality [11].

Our result when examining CVD mortality as the primary outcome are aligned with earlier research in the EPIC cohort, which identified a modest association between higher FSAm-NPS DI scores and increased mortality from circulatory diseases (13,246 events) [11]. In Italy and Spain, similar associations were reported in older population, between FSAm-NPS DI scores and cardiovascular mortalityin the Moli-sani study (792 events) [24] and ENRICA (140 events) cohort studies [14], respectively. However, this relationship was not evident in the Spanish SUN cohort (83 events) [12].

Interestingly, in our study no significant association was found with cancer-related mortality. Several factors may contribute to this finding including unmeasured bias, the heterogenetity of cancer subtypes and other factors related to the characteristics of this high cardiovascular risk population. Cancer risk or mortality was also evaluated in previous studies: in four of these studies [11, 12, 25, 26] a higher NS-NPM DI was associated with increased cancer incidence or mortality, ranging from a 7% to 144% increase, respectively. Notably, in a French prospective cohort study [27] with 46,864 women aged ≥ 35 years a 52% higher risk of breast cancer was found among those with the highest dietary score. However, some studies similar to ours, suggested only modest [26]^,^ or non-significant associations, albeit in the positive direction [25].

Additionally, the relatively low intake of red and processed meat and the high baseline consumption of fruits and vegetables across the cohort (characteristic of Mediterranean populations) may have resulted in a protective ‘floor effect,’ potentially attenuating the association with cancer mortality compared with cohorts from Northern Europe or North America.

The uNS-NPM DI reflects the quality of diet in terms of consumption of fruit and vegetables, and total energy, saturated fat, sugars, salt, fiber, and protein intake [5, 28]. In our study, those participants in the highest *u*NS-NPM DI quintile also consumed higher amounts of ultra-processed, and red meat and lower of healthy foods such as fruits, vegetables, and nuts, that have also been related to a reduced risk of cardiovascular/metabolic risk factors, chronic diseases and mortality [29–31]. Previous studies have also shown that *u*NS-NPM DI could discriminate adequately between healthy and unhealthy foods and has been validated for its discriminatory power to differentiate nutrients to consume or to be avoided [6, 32–34].

As a FOP labeling system designed to inform consumer choices, the Nutri-Score is intended to simplify the evaluation of food nutritional quality and has been validated to be objectively understandable at the point of purchase by Spanish consumers [35]. However, in a recent meta-analysis [36], the authors concluded that some of the weak and non-significant associations between the dietary indexes underlying FOP labels and health outcomes could partly be due to a fundamental limitation shared by all nutrient profiling systems: they assess only the nutritional quality and overlook the extent and purpose of food processing [37, 38]. The authors also suggested that front-of-pack nutrition labels should include warnings about the level of food processing [24, 39], as well as the presence of additives, pesticides, or contaminants released from packaging, since these factors may also play a role in developing chronic diseases [40–43]. Therefore, beyond the nutritional quality assessed by NS-NPM DI, other food diemensions must be taken into account in the future when evaluating diet and health outcomes.

Other limitations in our study must be acknowledged. First, although the FFQ used to measure dietary intake was validated and we calculated the cumulative average from baseline, some degree of misclassification bias may still be present. Second, due to its observational design, this study cannot establish a causal relationship between *u*NS-NPM DI and mortality. Third, despite robust adjustment, residual confounding from unmeasured variables is possible. Fourth, the median follow-up period of 6 years may be relatively short for evaluating long-term outcomes such as cancer mortality, which often involve lengthy latency periods. In addition, the number of cause-specific deaths—particularly for CVD and cancer—was modest, which may have limited statistical power to detect weaker associations.

Lastly, since the participants were older Mediterranean individuals from an intervention trial at high risk for cardiovascular disease, the findings may not be generalizable to other age groups or populations.

This study has several strengths, including the use of the uNS-NPM DI, a large and well-characterized Mediterranean cohort with long-term follow-up, and repeated dietary assessments collected through validated food frequency questionnaires, which enhance the accuracy of dietary exposure measurement. The analyses adjusted for a wide range of potential confounders, strengthening the validity of the findings. Moreover, sensitivity analyses excluding participants who died within the first one or two years of follow-up helped minimize reverse causality and addressed potential biases from baseline preclinical conditions, supporting the robustness of the observed associations.

In conclusion, among older adults at high cardiovascular risk, a higher *u*NS-NPM DI score—indicating lower diet quality assessed with the updated Nutri-Score algorithm - was prospectively associated with increased risks of all-cause, CVD, and other-cause but not cancer mortality. These findingssupport the utility of the uNS-NPM DI as a diet-quality indicator associated with mortality risk in this population.

## Supplementary Information

Below is the link to the electronic supplementary material.

Supplementary Material 1

Supplementary Material 2

## Data Availability

The datasets generated and analyzed during the current study are not expected to be made available outside the core research group, as neither participants’ consent forms nor ethics approval included permission for open access. However, the researchers will follow a controlled data sharing collaboration model, as in the informed consent participants agreed with a controlled collaboration with other investigators for research related to the project’s aims. Therefore, investigators who are interested in this study can contact the PREDIMED Steering Committee by sending a request letter to predimed-steering-commite@googlegroups.com. A data sharing agreement indicating the characteristics of the collaboration and data management will be completed for the proposals that are approved by the Steering Committee.
