# Supplementary material for: Mortality risk in relation to diet quality assessed by the 2023 nutri-score nutrient profiling model: a prospective analysis
Source: Eur J Nutr. 2026 Mar 24;65(3):102. doi: 10.1007/s00394-026-03946-4 (PMC13013389; doi:10.1007/s00394-026-03946-4)
Supplement: Supplementary file 1 — Supplementary Material 1 [file 394_2026_3946_MOESM1_ESM.docx]

**Title:** Mortality risk in relation to diet quality assessed by the 2023 Nutri-Score nutrient profiling model: A prospective analysis

Nadine Khoury^1,2,3a^ and Jose Cándido Fernández-Cao^4a^, Noushin Mohammadifard^5^, Miguel Ángel Martinez-González^3,6,7^, Dolores Corella^3,8^, Montserrat Fitó^3,9^, Ramón Estruch^10^, Lucas Tojal-Sierra^3,11^, Enrique Gómez Gracias^3,12^, Miquel Fiol^3,13^, José Lapetra^3,14^, Lluís Serra-Majem^3,15^, Xavier Pintó^3,16^, Zenaida Vázquez-Ruiz^3,5^, Jose V. Sorli^3,8^, Helmut Schröder^8,17^, Jordi Salas-Salvadó^1,2,3#^, Nancy Babio^1,2,3#^.

a: These authors contributed equally to this work.

#: Correspondence: E-mail addresses: nancy.babio@urv.cat (Nancy Babio), jordi.salas@urv.cat (Jordi Salas-Salvadó).

**Affiliations:**

1. Institut d'Investigació Sanitària Pere Virgil i (IISPV), Grup Alimentació, Nutrició, esenvolupament i salut Mental, Reus 43204, Spain.
2. Universitat Rovira i Virgili, Departament de Bioquímica i Biotecnologia, Alimentació, Nutrició, Desenvolupament i Salut Mental ANUT-DSM, Reus 43204, Spain.
3. Centro de Investigación Biomédica en Red Fisiopatología de La Obesidad y Nutrición (CIBEROBN), Instituto de Salud Carlos III, Madrid 28029, Spain.
4. Department of Nutrition and Dietetics, Faculty of Health Sciences, University of Atacama, Copiapó, Chile.
5. Isfahan Cardiovascular Research Center, Cardiovascular Research Institute, Isfahan University of Medical Sciences, Isfahan, Iran.
6. Department of Preventive Medicine and Public Health, School of Medicine, University of Navarra, Pamplona, Spain.
7. Department of Nutrition, Harvard T.H. Chan School of Public Health, Boston, MA, United States.
8. Department of Preventive Medicine and Public Health, School of Medicine, University of Valencia, Valencia, Spain.
9. Unit of Cardiovascular Risk and Nutrition, Institut Hospital del Mar de Investigaciones Médicas Municipal d'Investigació Médica (IMIM), Barcelona 08003, Spain.
10. Department of Internal Medicine, Institut d'Investigacions Biomèdiques August Pi Sunyer (IDIBAPS), Hospital Clinic, University of Barcelona, Barcelona 08036, Spain.
11. Department of Cardiology, Hospital Universitario de Álava, Vitoria, Spain.
12. Preventive Medicine and Public Health, University of Malaga, Malaga 29071, Spain.
13. Bioaraba Health Research Institute, Osakidetza Basque Health Service, Araba University Hospital, University of the Basque Country UPV/EHU, Vitoria-Gasteiz 01006, Spain.
14. Department of Family Medicine, Research Unit, Distrito Sanitario Atención Primaria Sevilla, Sevilla 41009, Spain.
15. Research Institute of Biomedical and Health Sciences (IUIBS), University of Las Palmas de Gran Canaria & Centro Hospitalario Universitario Insular Materno Infantil (CHUIMI), Canarian Health Service, Las Palmas de Gran Canaria 35016, Spain.
16. Lipid and Vascular Risk Unit, Internal Medicine Service, Hospital Universitario de Bellvitge, L'Hospitalet de Llobregat, Spain.
17. Centro de Investigación Biomédica en Red Epidemiología y Salud Pública (CIBEResp), Instituto de Salud Carlos III, Madrid 28029, Spain.

**Supplementary Material File S1. The updated Nutri-Score algorithms**

|  | **Nutri-Score Algorithm for general food** | | | | | | |
| --- | --- | --- | --- | --- | --- | --- | --- |
| Points | **Unfavorable components** | | | | **Favorable components** | | |
|  | Energy  (kJ per 100g) | Sugars  (g per 100g) | Saturated fat  (g per 100g) | Salt  (g per 100g) | Protein* (g per 100g) | Fiber  (g per 100g) | Fruit, vegetables, legumes (%) |
| 0 | ≤ 335 | ≤ 3.4 | ≤ 1.0 | ≤ 0.2 | ≤ 2.4 | ≤ 3.0 | ≤ 40 |
| 1 | > 335 | > 3.4 | > 1.0 | > 0.2 | > 2.4 | > 3.0 | > 40 |
| 2 | > 670 | > 6.8 | > 2.0 | > 0.4 | > 4.8 | > 4.1 | > 60 |
| 3 | > 1005 | > 10 | > 3.0 | > 0.6 | > 7.2 | > 5.2 | - |
| 4 | > 1340 | > 14 | > 4.0 | > 0.8 | > 9.6 | > 6.3 | - |
| 5 | > 1675 | > 17 | > 5.0 | > 1.0 | > 12 | > 7.4 | > 80 |
| 6 | > 2010 | > 20 | > 6.0 | > 1.2 | > 14 |  |  |
| 7 | > 2345 | > 24 | > 7.0 | > 1.4 | > 17 |  |  |
| 8 | > 2680 | > 27 | > 8.0 | > 1.6 |  |  |  |
| 9 | > 3015 | > 31 | > 9.0 | > 1.8 |  |  |  |
| 10 | > 3350 | > 34 | > 10 | > 2.0 |  |  |  |
| 11 |  | > 37 |  | > 2.2 |  |  |  |
| 12 |  | > 41 |  | > 2.4 |  |  |  |
| 13 |  | > 44 |  | > 2.6 |  |  |  |
| 14 |  | > 48 |  | > 2.8 |  |  |  |
| 15 |  | > 51 |  | > 3.0 |  |  |  |
| 16 |  |  |  | > 3.2 |  |  |  |
| 17 |  |  |  | > 3.4 |  |  |  |
| 18 |  |  |  | > 3.6 |  |  |  |
| 19 |  |  |  | > 3.8 |  |  |  |
| 20 |  |  |  | > 4.0 |  |  |  |

*Red meat products are given maximum 2 protein points.

| **Nutri-Score total points for general food** | **Class** | **Color** |
| --- | --- | --- |
| ≤ 0 | A | Dark green |
| 1 to 2 | B | Light green |
| 3 to 10 | C | Yellow |
| 11 to 18 | D | Light orange |
| ≥ 19 | E | Dark orange |

|  | **Nutri-Score total points for fats, oils, nuts and seeds** | | | | | | |
| --- | --- | --- | --- | --- | --- | --- | --- |
| Points | **Unfavorable components** | | | | **Favorable components** | | |
|  | Energy from saturated fat  (kJ per 100g) * | Sugars  (g per 100g) | Saturated fat/total fat (%) | Salt  (g per 100g) | Protein (g per 100g) | Fiber  (g per 100g) | Fruit, vegetables, legumes (%) |
| 0 | ≤ 120 | ≤ 3.4 | < 10 | ≤ 0.2 | ≤ 2.4 | ≤ 3.0 | ≤ 40 |
| 1 | > 120 | > 3.4 | < 16 | > 0.2 | > 2.4 | > 3.0 | > 40 |
| 2 | > 240 | > 6.8 | < 22 | > 0.4 | > 4.8 | > 4.1 | > 60 |
| 3 | > 360 | > 10 | < 28 | > 0.6 | > 7.2 | > 5.2 | - |
| 4 | > 480 | > 14 | < 34 | > 0.8 | > 9.6 | > 6.3 | - |
| 5 | > 600 | > 17 | < 40 | > 1.0 | > 12 | > 7.4 | > 80 |
| 6 | > 720 | > 20 | < 46 | > 1.2 | > 14 |  |  |
| 7 | > 840 | > 24 | < 52 | > 1.4 | > 17 |  |  |
| 8 | > 960 | > 27 | < 58 | > 1.6 |  |  |  |
| 9 | > 1080 | > 31 | < 64 | > 1.8 |  |  |  |
| 10 | > 1200 | > 34 | ≥ 64 | > 2.0 |  |  |  |
| 11 |  | > 37 |  | > 2.2 |  |  |  |
| 12 |  | > 41 |  | > 2.4 |  |  |  |
| 13 |  | > 44 |  | > 2.6 |  |  |  |
| 14 |  | > 48 |  | > 2.8 |  |  |  |
| 15 |  | > 51 |  | > 3.0 |  |  |  |
| 16 |  |  |  | > 3.2 |  |  |  |
| 17 |  |  |  | > 3.4 |  |  |  |
| 18 |  |  |  | > 3.6 |  |  |  |
| 19 |  |  |  | > 3.8 |  |  |  |
| 20 |  |  |  | > 4.0 |  |  |  |

*Energy from saturated fat = saturated fat (g per 100 grams) x 37

| **Nutri-Score total points for fats, oils, nuts and seeds** | **Class** | **Color** |
| --- | --- | --- |
| ≤ -6 | A | Dark green |
| -5 to 2 | B | Light green |
| 3 to 10 | C | Yellow |
| 11 to 18 | D | Light orange |
| ≥ 19 | E | Dark orange |

|  | **Nutri-Score total points for beverages** | | | | | | | |
| --- | --- | --- | --- | --- | --- | --- | --- | --- |
| Points | **Unfavorable components** | | | | | **Favorable components** | | |
|  | Energy  (kJ per 100ml) | Sugars  (g per 100ml) | Saturated fat  (g per 100ml) | Salt  (g per 100ml) | Non-nutritive sweeteners  (absence/presence) | Protein  (g per 100ml) | Fiber  (g per 100ml) | Fruit, vegetables, legumes (%) |
| 0 | ≤ 30 | ≤ 0.5 | ≤ 1.0 | ≤ 0.2 |  | ≤ 1.2 | ≤ 3.0 | ≤ 40 |
| 1 | ≤ 90 | ≤ 2.0 | > 1.0 | > 0.2 |  | > 1.2 | > 3.0 | - |
| 2 | ≤ 150 | ≤ 3.5 | > 2.0 | > 0.4 |  | > 1.5 | > 4.1 | > 40 |
| 3 | ≤ 210 | ≤ 5.0 | > 3.0 | > 0.6 |  | > 1.8 | > 5.2 | - |
| 4 | ≤ 240 | ≤ 6.0 | > 4.0 | > 0.8 | Presence | > 2.1 | > 6.3 | > 60 |
| 5 | ≤ 270 | ≤ 7.0 | > 5.0 | > 1.0 |  | > 2.4 | > 7.4 | - |
| 6 | ≤ 300 | ≤ 8.0 | > 6.0 | > 1.2 |  | > 2.7 |  | > 80 |
| 7 | ≤ 330 | ≤ 9.0 | > 7.0 | > 1.4 |  | > 3.0 |  |  |
| 8 | ≤ 360 | ≤ 10 | > 8.0 | > 1.6 |  |  |  |  |
| 9 | ≤ 390 | ≤ 11 | > 9.0 | > 1.8 |  |  |  |  |
| 10 | > 390 | > 11 | > 10 | > 2.0 |  |  |  |  |
|  |  |  |  | > 2.2 |  |  |  |  |
|  |  |  |  | > 2.4 |  |  |  |  |
|  |  |  |  | > 2.6 |  |  |  |  |
|  |  |  |  | > 2.8 |  |  |  |  |
|  |  |  |  | > 3.0 |  |  |  |  |
|  |  |  |  | > 3.2 |  |  |  |  |
|  |  |  |  | > 3.4 |  |  |  |  |
|  |  |  |  | > 3.6 |  |  |  |  |
|  |  |  |  | > 3.8 |  |  |  |  |
|  |  |  |  | > 4.0 |  |  |  |  |

| **Nutri-Score total points for beverages** | **Class** | **Color** |
| --- | --- | --- |
| Water* | A | Dark green |
| ≤ 2 | B | Light green |
| 3 to 6 | C | Yellow |
| 7 to 9 | D | Light orange |
| ≥ 10 | E | Dark orange |

*Plain water was automatically given Nutri-Score class A.

**Supplementary Material File S2.** Interactions between the *u*NS-NPM DI (1-SD increment) and other covariates in associations with mortality risk, and corresponding subgroup analyses, multivariable Cox proportional hazards regression models, PREDIMED study.

|  | **P-interaction** | **Events** | **HR (95% CI)**  **For 1-SD increment** | **P-value** |
| --- | --- | --- | --- | --- |
| **All-cause mortality** |  |  |  |  |
| **Sex** | 0.69 |  |  |  |
| Male |  | 259/3067 | 1.10 (0.96 to 1.26) | 0.151 |
| Female |  | 165/4143 | 1.19 (1.02 to 1.3) | 0.027 |
| **Education level** | 0.55 |  |  |  |
| Primary |  | 329/5061 | 1.11 (0.99 to 1.24) | 0.083 |
| Secondary |  | 53/1095 | 1.26 (0.91 to 1.75) | 0.167 |
| University |  | 42/514 | 1.30 (0.92 to 1.84) | 0.132 |
| **BMI** | 0.335 |  |  |  |
| < 25 kg/m² |  | 234/3827 | 1.14 (0.99 to 1.31) | 0.066 |
| ≥ 25 kg/m² |  | 190/3383 | 1.13 (0.97 to 1.32) | 0.111 |
| **Age** | 0.436 |  |  |  |
| < 67 y |  | 120/3494 | 1.25 (1.05 to 1.48) | 0.010 |
| ≥ 67 y |  | 304/3716 | 1.11 (0.98 to 1.26) | 0.100 |
| **Adherence to Mediterranean diet (MEDAS score 0-14 points)** | 0.236 |  |  |  |
| <7 |  | 24//3029 | 1.21 (1.06 to 1.38) | 0.005 |
| ≥7 |  | 175/4181 | 1.03 (0.88 to 1.21) | 0.667 |
| **UPF consumption (Cumulative average, energy-adjusted, g/day)** | 0.107 |  |  |  |
| <261 |  | 202/3605 | 1.16 (0.98 to 1.38) | 0.081 |
| ≥261 |  | 222/3605 | 1.13 (0.97 to 1.31) | 0.105 |
| **CVD mortality** |  |  |  |  |
| **Sex** | 0.961 |  |  |  |
| Male |  | 62/3067 | 1.32 (1.00 to 1.73) | 0.048 |
| Female |  | 41/4143 | 1.49 (1.13 to 1.98) | 0.005 |
| **Education level** | 0.734 |  |  |  |
| Primary |  | 78/5601 | 1.37 (1.10 to 1.71) | 0.005 |
| Secondary |  | 15/1095 | 1.85 (1.09 to 3.12) | 0.021 |
| University |  | 10/514 | 0.44 (0.12 to 1.60) | 0.211 |
| **BMI** | 0.604 |  |  |  |
| < 25 kg/m² |  | 51/3827 | 1.45 (1.08 to 1.94) | 0.013 |
| ≥ 25 kg/m² |  | 52/3383 | 1.28 (0.96 to 1.71) | 0.097 |
| **Age** | 0.783 |  |  |  |
| < 67 y |  | 26/3494 | 1.34 (0.95 to 1.91) | 0.099 |
| ≥ 67 y |  | 77/3716 | 1.43 (1.12 to 1.82) | 0.004 |
| **Adherence to Mediterranean diet (MEDAS score 0-14 points)** | 0.869 |  |  |  |
| <7 |  | 60/3029 | 1.58 (1.23 to 2.03) | <0.001 |
| ≥7 |  | 43/4181 | 1.19 (0.86 to 1.66) | 0.280 |
| **UPF consumption (Cumulative average, energy-adjusted, g/day)** | 0.825 |  |  |  |
| <261 |  | 45/3605 | 1.58 (1.15 to 2.17) | 0.005 |
| ≥261 |  | 58/3605 | 1.36 (1.03 to 1.79) | 0.031 |
| **Cancer mortality** |  |  |  |  |
| **Sex** | 0.935 |  |  |  |
| Male |  | 108/3067 | 0.96 (0.77 to 1.19) | 0.692 |
| Female |  | 61/4143 | 1.05 (0.79 to 1.41) | 0.722 |
| **Education level** | 0.159 |  |  |  |
| Primary |  | 133/5601 | 0.96 (0.79 to 1.18) | 0.733 |
| Secondary |  | 22/1095 | 0.92 (0.54 to 1.59) | 0.777 |
| University |  | 14/514 | 1.62 (0.87 to 3.04) | 0.129 |
| **BMI** | 0.124 |  |  |  |
| < 25 kg/m² |  | 93/3827 | 1.04 (0.82 to 1.32) | 0.735 |
| ≥ 25 kg/m² |  | 76/3383 | 0.93 (0.71 to 1.23) | 0.625 |
| **Age** | 0.426 |  |  |  |
| < 67 y |  | 59/3494 | 1.09 (0.84 to 1.41) | 0.514 |
| ≥ 67 y |  | 110/3716 | 0.95 (0.75 to 1.19) | 0.671 |
| **Adherence to Mediterranean diet (MEDAS score 0-14 points)** | 0.129 |  |  |  |
| <7 |  | 94/3029 | 1.05 (0.80 to 1.37) | 0.709 |
| ≥7 |  | 75/4181 | 0.91 (0.73 to 1.13) | 0.385 |
| **UPF consumption (Cumulative average, energy-adjusted, g/day)** | 0.179 |  |  |  |
| <261 |  | 75/3605 | 0.95 (0.69 to 1.30) | 0.760 |
| ≥261 |  | 94/3605 | 0.98 (0.76 to 1.26) |  |
| **Other causes of mortality** |  |  |  |  |
| **Sex** | 0.764 |  |  |  |
| Male |  | 89/3067 | 1.13 (0.89 to 1.44) | 0.308 |
| Female |  | 63/4143 | 1.13 (0.89 to 1.42) | 0.314 |
| **Education level** | 0.648 |  |  |  |
| Primary |  | 118/5601 | 1.09 (0.90 to 1.33) | 0.358 |
| Secondary |  | 16/1095 | 1.45 (0.74 to 2.85) | 0.281 |
| University |  | 18/514 | 1.12 (0.69 to 1.85) | 0.639 |
| **BMI** | 0.990 |  |  |  |
| < 25 kg/m² |  | 90/3827 | 1.11 (0.88 to 1.39) | 0.384 |
| ≥ 25 kg/m² |  | 62/3383 | 1.19 (0.95 to 1.49) | 0.133 |
| **Age** | 0.340 |  |  |  |
| < 67 y |  | 35/3494 | 1.44 (1.12 to 1.87) | 0.005 |
| ≥ 67 y |  | 117/3716 | 1.08 (0.89 to 1.33) | 0.427 |
| **Adherence to Mediterranean diet (MEDAS score 0-14 points)** | 0.713 |  |  |  |
| <7 |  | 95/3029 | 1.15 (0.96 to 1.38) | 0.123 |
| ≥7 |  | 57/4181 | 1.06 (0.79 to 1.41) | 0.676 |
| **UPF consumption (Cumulative average, energy-adjusted, g/day)** | 0.459 |  |  |  |
| <261 |  | 82/3605 | 1.16 (0.88 to 1.53) | 0.272 |
| ≥261 |  | 70/3605 | 1.14 (0.89 to 1.44) | 0.295 |

A higher uNS-NPS DI indicates a lower nutritional quality of the foods consumed

Abbreviations: DI, Dietary Index; uNS-NPS, updated Nutrient Profiling Model underlying the Nutri-Score; SD, standard deviation; BMI: body mass index; UPF: ultra-processed food.

Multivariable Cox proportional hazards regression minimally adjusted model: adjusted for age (years, continuous) and sex (male, female). Fully-adjusted model: further adjusted for total energy intake (Kcal, continuous), intervention group (Olive oil, nuts, low fat diet), education level (primary, secondary, university), smoking status (never, former, current), physical activity (METS min/day, continuous), BMI (normal weight and overweight/ obesity), alcohol consumption (g/day, continuous) family history of cancer (yes/no), diabetes (yes/no), hypertension (yes/no) and hypercholesterolemia (yes/no).

**Supplementary Material File S3.** Association between *u*NS-NPM DI and cause-specific and all-cause mortality; multivariable Cox proportional hazards regression models excluding participants experiencing a mortality event within the first 1-year of follow-up (n=7,175).

|  | *u*NS-NPM DI in quintiles  Hazard ratios (95% CI) | | | | |  |
| --- | --- | --- | --- | --- | --- | --- |
|  | **Q1**  **n=1,438** | **Q2**  **n=1,437** | **Q3**  **n=1,435** | **Q4**  **n=1,432** | **Q5**  **n=1,433** | **P-trend** |
| All-cause mortality  *394 cases/ 42442 py* | 61 cases | 73 cases | 87 cases | 76 cases | 97 cases |  |
| Mortality rate (per 1000)* | 6.92 | 8.61 | 10.25 | 9.11 | 11.65 |  |
| Minimally-adjusted | 1 (ref.) | 1.19 (0.85 to 1.67) | **1.41 (1.01 to 1.94)** | 1.17 (0.84 to 1.64) | **1.52 (1.10 to 2.09)** | **0.035** |
| Fully-adjusted | 1 (ref.) | 1.21 (0.86 to 1.69) | **1.48 (1.06 to 2.05)** | 1.23 (0.87 to 1.75) | **1.64 (1.17 to 2.30)** | **0.013** |
| CVD mortality  *94 cases/* *42442 py* | 7 cases | 8 cases | 26 cases | 26 cases | 27 cases |  |
| Mortality rate (per 1000)* | 0.79 | 0.94 | 3.06 | 3.12 | 3.24 |  |
| Minimally-adjusted | 1 (ref.) | 1.08 (0.39 to 2.98) | **3.43 (1.47 to 8.03)** | **3.24 (1.38 to 7.63)** | **3.58 (1.53 to 8.37)** | **0.006** |
| Fully-adjusted | 1 (ref.) | 1.00 (0.37 to 2.73) | **3.45 (1.46 to 8.13)** | **3.07 (1.26 to 7.48)** | **3.25 (1.28 to 8.25)** | **0.007** |
| Cancer *mortality*  *157 cases/* *42442 py* | 31 cases | 35 cases | 30 cases | 27 cases | 34 cases |  |
| Mortality rate (per 1000)* | 3.52 | 4.13 | 3.53 | 3.24 | 4.08 |  |
| Minimally-adjusted | 1 (ref.) | 1.12 (0.69 to 1.81) | 0.94 (0.57 to 1.55) | 0.82 (0.49 to 1.37) | 1.01 (0.62 to 1.65) | 0.946 |
| Fully-adjusted | 1 (ref.) | 1.12 (0.70 to 1.80) | 0.95 (0.58 to 1.58) | 0.85 (0.50 to 1.43) | 1.06 (0.62 to 1.79) | 0.951 |
| Other causes of mortality  *143 cases/ 42442 py* | 23 cases | 30 cases | 31 cases | 23 cases | 36 cases |  |
| Mortality rate (per 1000)* | 2.95 | 3.65 | 3.77 | 3.11 | 4.56 |  |
| Minimally-adjusted | 1 (ref.) | 1.35 (0.79 to 2.33) | 1.36 (0.79 to 2.34) | 0.98 (0.55 to 1.75) | 1.54 (0.91 to 2.59) | 0.259 |
| Fully-adjusted | 1 (ref.) | 1.49 (0.86 to 2.59) | 1.55 (0.90 to 2.68) | 1.13 (0.61 to 2.09) | **1.85 (1.13 to 3.36)** | 0.101 |

Abbreviations: *u*NS-NPM DI: updated Nutri-Score Nutrient Profiling Model Dietary Index; CVD: cardiovascular disease; CI: Confidence interval; py: person-years.

* failures/person-time (per 1000)

Multivariable Cox proportional hazards regression minimally adjusted model: adjusted for age (years, continuous) and sex (male, female). Fully-adjusted model: further adjusted for total energy intake (Kcal, continuous), intervention group (Olive oil, nuts, low fat diet), education level (primary, secondary, university), smoking status (never, former, current), physical activity (METS min/day, continuous), BMI (normal weight and overweight/obesity), alcohol consumption (g/day, continuous) family history of cancer (yes/no), diabetes (yes/no), hypertension (yes/no) and hypercholesterolemia (yes/no).

**Supplementary Material File S4.** Association between *u*NS-NPM DI and cause-specific and all-cause mortality; multivariable Cox proportional hazards regression models excluding participants experiencing a mortality event within the first 2-year of follow-up (n=7,112).

|  | *u*NS-NPM DI in quintiles  Hazard ratios (95% CI) | | | | |  |
| --- | --- | --- | --- | --- | --- | --- |
|  | **Q1**  **n=1,428** | **Q2**  **n=1,430** | **Q3**  **n=1,425** | **Q4**  **n=1,415** | **Q5**  **n=1,414** | **P-trend** |
| All-cause mortality  *351 cases/ 42344 py* | 51 cases | 68 cases | 80 cases | 64 cases | 88 cases |  |
| Mortality rate (per 1000)* | 5.79 | 8.03 | 9.44 | 7.69 | 10.61 |  |
| Minimally-adjusted | 1 (ref.) | 1.33 (0.93 to 1.91) | **1.55 (1.09 to 2.19)** | 1.18 (0.82 to 1.71) | **1.66 (1.18 to 2.34)** | **0.011** |
| Fully-adjusted | 1 (ref.) | 1.34 (0.94 to 1.92) | **1.61 (1.14 to 2.29)** | 1.22 (0.84 to 1.78) | **1.76 (1.22 to 2.53)** | **0.005** |
| CVD mortality  *85 cases/* 42344 *py* | 5 cases | 8 cases | 24 cases | 23 cases | 25 cases |  |
| Mortality rate (per 1000)* | 0.56 | 0.94 | 2.83 | 2.77 | 3.01 |  |
| Minimally-adjusted | 1 (ref.) | 1.50 (0.49 to 4.56) | **4.39 (1.66 to 11.6)** | **3.97 (1.50 to 10.4)** | **4.64 (1.76 to 12.2)** | **0.002** |
| Fully-adjusted | 1 (ref.) | 1.35 (0.45 to 4.07) | **4.19 (1.57 to 11.2)** | **3.43 (1.24 to 9.49)** | **3.76 (1.30 to 10.8** | **0.004** |
| Cancer *mortality*  *137 cases/* *42344 py* | 27 cases | 31 cases | 26 cases | 23 cases | 30 cases |  |
| Mortality rate (per 1000)* | 3.07 | 3.65 | 3.07 | 3.77 | 3.62 |  |
| Minimally-adjusted | 1 (ref.) | 1.15 (0.69 to 1.90) | 0.94 (0.55 to 1.61) | 0.81 (0.46 to 1.41) | 1.05 (0.62 to 1.77) | 0.933 |
| Fully-adjusted | 1 (ref.) | 1.16 (0.69 to 1.91) | 0.96 (0.56 to 1.65) | 0.85 (0.48 to 1.50) | 1.12 (0.64 to 1.96) | 0.990 |
| Other causes of mortality  *129 cases/ 42344 py* | 19 cases | 29 cases | 30 cases | 18 cases | 33 cases |  |
| Mortality rate (per 1000)* | 2.95 | 3.65 | 3.77 | 3.11 | 4.56 |  |
| Minimally-adjusted | 1 (ref.) | 1.59 (0.89 to 2.85) | 1.60 (0.89 to 2.85) | 0.93 (0.45 to 1.77) | 1.70 (0.97 to 2.99) | 0.109 |
| Fully-adjusted | 1 (ref.) | 1.77 (0.99 to 3.18) | **1.84 (1.04 to 3.27)** | 1.07 (0.54 to 2.11) | **2.18 (1.22 to 3.89)** | **0.035** |

Abbreviations: *u*NS-NPM DI: updated Nutri-Score Nutrient Profiling Model Dietary Index; CVD: cardiovascular disease; CI: Confidence interval; py: person-years.

* failures/person-time (per 1000)

Multivariable Cox proportional hazards regression minimally adjusted model: adjusted for age (years, continuous) and sex (male, female). Fully-adjusted model: further adjusted for total energy intake (Kcal, continuous), intervention group (Olive oil, nuts, low fat diet), education level (primary, secondary, university), smoking status (never, former, current), physical activity (METS min/day, continuous), BMI (normal weight and overweight/obesity), alcohol consumption (g/day, continuous) family history of cancer (yes/no), diabetes (yes/no), hypertension (yes/no) and hypercholesterolemia (yes/no).

**Supplementary Material File S5.** Association between uNS-NPM DI and cause-specific mortality in Fine–Gray competing-risks models (n=7,212).

| **uNS-NPM DI Quintile** | **Cases (n)** | **Mortality rate* (per 1000py)** | **Fully-adjusted SHR (95% CI)** |
| --- | --- | --- | --- |
| **CVD mortality** |  |  |  |
| Q1 (n=1,443) | 7 | 0.79 | 1 (ref.) |
| Q2 (n=1,442) | 8 | 0.94 | 1.03 (0.37–2.82) |
| Q3 (n=1,443) | 30 | 3.53 | 4.08 (1.79–9.29) |
| Q4 (n=1,442) | 29 | 3.47 | 3.71 (1.62–8.52) |
| Q5 (n=1,442) | 29 | 3.48 | 3.64 (1.55–8.55) |
| P-trend | – | – | 0.001 |
| **Cancer mortality** |  |  |  |
| Q1 (n=1,443) | 32 | 3.63 | 1 (ref.) |
| Q2 (n=1,442) | 38 | 4.48 | 1.15 (0.72–1.84) |
| Q3 (n=1,443) | 33 | 3.88 | 0.97 (0.59–1.58) |
| Q4 (n=1,442) | 29 | 3.47 | 0.82 (0.49–1.38) |
| Q5 (n=1,442) | 37 | 4.44 | 1.01 (0.62–1.63) |
| P-trend | – | – | 0.904 |
| **Other-cause mortality** |  |  |  |
| Q1 (n=1,443) | 26 | 2.95 | 1 (ref.) |
| Q2 (n=1,442) | 31 | 3.65 | 1.26 (0.74–2.13) |
| Q3 (n=1,443) | 32 | 3.77 | 1.29 (0.77–2.18) |
| Q4 (n=1,442) | 26 | 3.11 | 0.99 (0.56–1.75) |
| Q5 (n=1,442) | 38 | 4.56 | 1.55 (0.93–2.59) |
| P-trend | – | – | 0.321 |

SHR: subdistribution hazard ratio from Fine–Gray competing-risks regression.

Models are fully-adjusted for age (years, continuous), sex (male, female), total energy intake (Kcal, continuous), intervention group (Olive oil, nuts, low fat diet), education level (primary, secondary, university), smoking status (never, former, current), physical activity (METS min/day, continuous), BMI (normal weight and overweight/obesity), alcohol consumption (g/day, continuous) family history of cancer (yes/no), diabetes (yes/no), hypertension (yes/no) and hypercholesterolemia (yes/no).

Mortality rate is crude, calculated as cases / person-years × 1000.

P-trend calculated by modeling quintiles as a continuous variable in the Fine–Gray model.

**Supplementary Material File S5.** Association between uNS-NPM DI at baseline (with 1 SD increment) and cause-specific and all-cause mortality


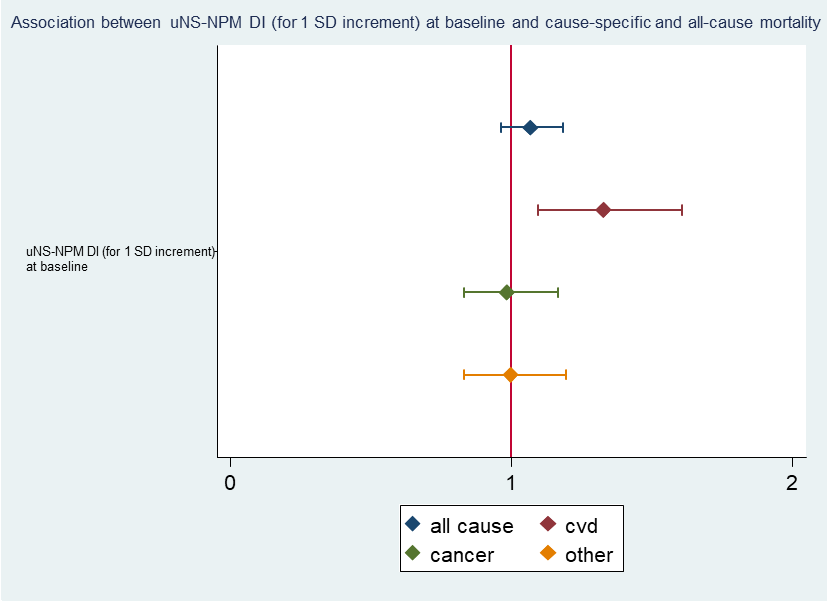


Association between *u*NS-NPM DI at baseline (for 1 SD increment) and cause-specific and all-cause mortality; multivariable Cox proportional hazards regression models (n=7,212).

Abbreviations: *u*NS-NPM DI: updated Nutri-Score Nutrient Profiling Model Dietary Index; CVD: cardiovascular disease; HR: Hazard Ratios; CI: Confidence interval; SD: Standard Deviation

Multivariable Cox proportional hazards regression minimally adjusted model: adjusted for age (continuous) and sex (male, female). Fully-adjusted model: further adjusted for total energy intake (Kcal, continuous), intervention group (Olive oil, nuts, low fat diet), education level (Primary, Secondary, University), smoking status (Never, Former, Current), physical activity (METS min/day, continuous), BMI (normal weight and overweight/obesity), alcohol consumption (g/day, continuous) family history of cancer, diabetes, hypertension and hypercholesterolemia (yes, no).
